# P-2057. Social Determinants of Health (SDOH) are Associated with Non-Intravascular Device (IVAD) Related Adverse Events (AE) in Patients Treated with Outpatient Parenteral Antimicrobial Therapy (OPAT)

**DOI:** 10.1093/ofid/ofaf695.2221

**Published:** 2026-01-11

**Authors:** Colin Samoriski, Mike Sportiello, Alexandra Yamshchikov

**Affiliations:** University of Rochester, Rochester, NY; Emory University, Atlanta, GA; University of Rochester School of Medicine and Dentistry, Rochester, NY

## Abstract

**Background:**

SDOH contribute to morbidity and mortality in health care settings including OPAT. Few risk stratification models for OPAT include evaluation of SDOH as contributing drivers of inequitable outcomes.

**Figure 1.**
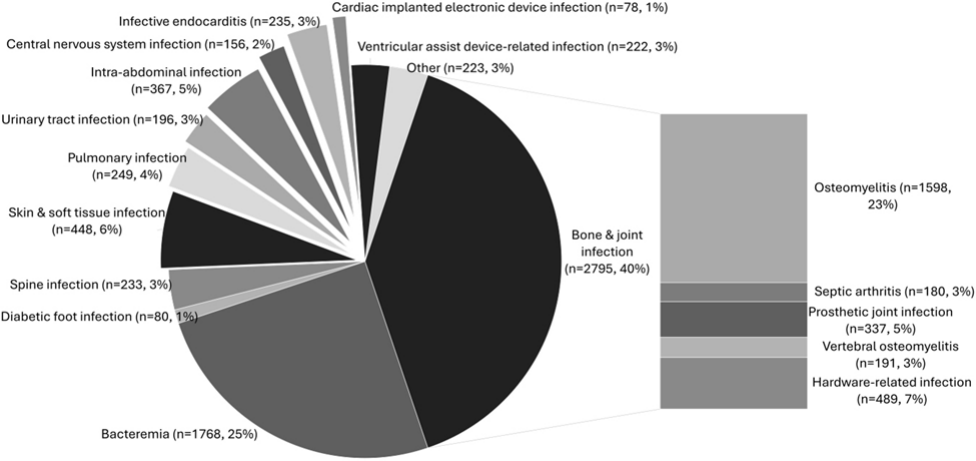
Indications for OPAT (N=5283)

**Table 1.**
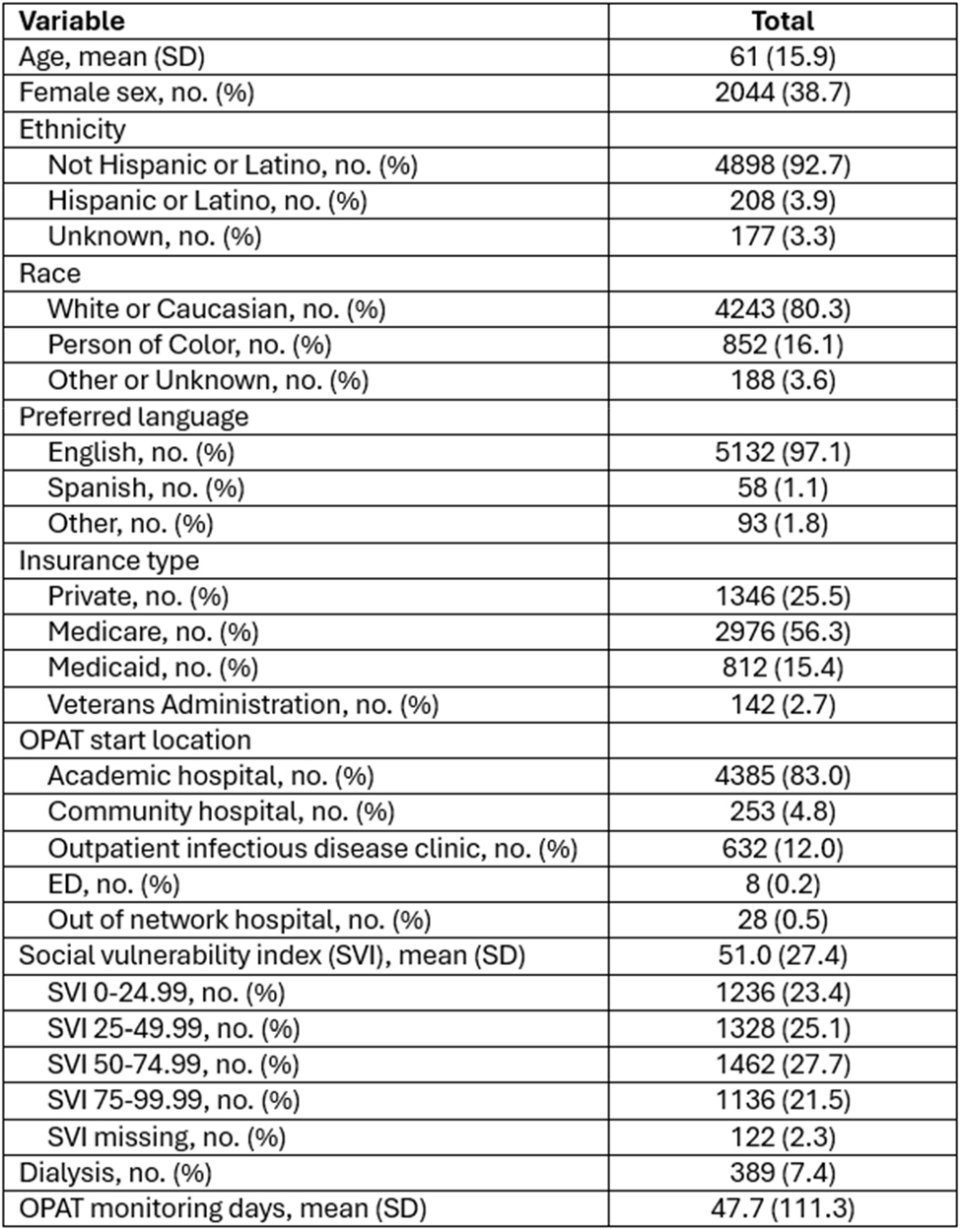
Patient Characteristics

**Methods:**

Using a previously reported OPAT-specific bundle of modifications to an Epic® electronic health record (EHR), we developed an EHR-based electronic SQL report evaluating OPAT-related AE within an OPAT program at a large academic medical center. The impact of insurance status, OPAT start location, race/ethnicity, preferred language, social vulnerability index (SVI) on non-IVAD related AE, including nephrotoxicity (defined as >0.3 increase or >1.5 times increase in baseline creatinine), hospital readmissions, and emergency department (ED) utilization while on OPAT or within 30 days of conclusion, and mortality within 1 year of OPAT start was assessed by logistic regression using R v4.4.3.

**Table 2.**
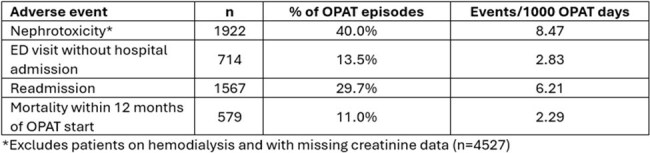
Rates of Electronically Captured Non-Intravascular Device Related OPAT Adverse Events

**Table 3.**
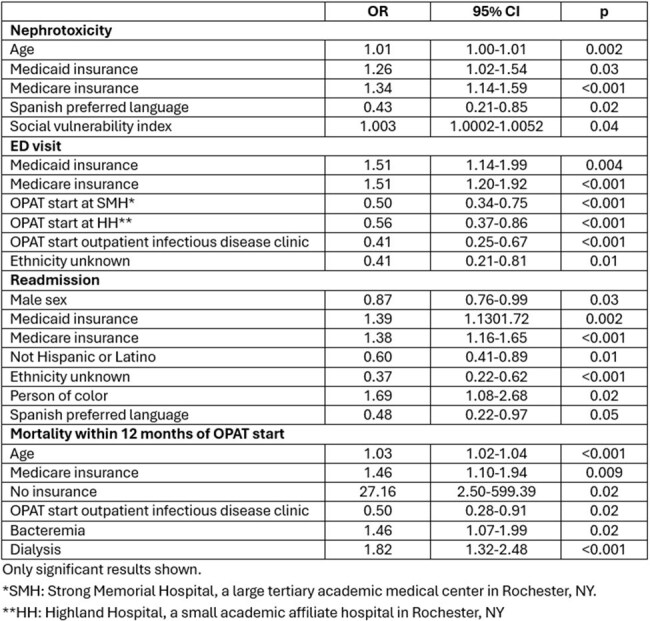
Association Between Treatment-Related Factors and Social Determinants with Electronically Captured Non-Intravascular Device Related OPAT Complications

**Results:**

A total of 5283 patients (Table 1) with varying indications (Figure 1) for OPAT were identified from 10/10/2018-12/31/2024 . Nephrotoxicity was the most frequently observed adverse event (Table 2). Patients with public insurance were more likely to experience nephrotoxicity, ED use, and readmission (Table 3). Identifying as a person of color was associated with higher (OR 1.70, CI 1.08-2.69), while reported ethnicity of not Hispanic/Latino with lower (OR 0.60, CI 0.41-0.89) rates of readmission. Spanish as preferred language was associated with lower rates of nephrotoxicity (OR 0.43, CI 0.21-0.85) and readmission (OR 0.48, CI 0.22-0.97). Starting OPAT at academic hospitals as compared to rural community hospitals was associated with reduced ED use. Mortality was associated with age (OR 1.03, CI 1.02-1.04), receipt of dialysis (OR 1.82, CI 1.32-2.48), bacteremia (OR 1.46, CI 1.07-1.99), Medicare (OR 1.46, CI 1.10-1.94), and lack of insurance (OR 27.16, CI 2.50-599.39).

**Conclusion:**

Social determinants were associated with multiple AE in patients treated with OPAT, although more studies are needed to evaluate the interplay of medical or treatment related factors, and social determinants of AE. Incorporating SDOH into longitudinal monitoring of OPAT-related AE may help identify patients for additional monitoring or intervention to mitigate inequitable outcomes.

**Disclosures:**

All Authors: No reported disclosures

